# Dynamic touch reduces physiological arousal in preterm infants: A role for c-tactile afferents?

**DOI:** 10.1016/j.dcn.2019.100703

**Published:** 2019-08-21

**Authors:** Andrea Manzotti, Francesco Cerritelli, Jorge E. Esteves, Gianluca Lista, Erica Lombardi, Simona La Rocca, Alberto Gallace, Francis P. McGlone, Susannah C. Walker

**Affiliations:** a RAISE Lab, Clinical-Based Human Research Department, Foundation COME Collaboration, Pescara, Italy; b Division of Neonatology, “V. Buzzi” Children’s Hospital, ASST-FBF-Sacco, Milan, Italy; c Research Department, SOMA, Istituto Osteopatia Milano, Milan, Italy; d Gulf National Centre, Foundation COME Collaboration, Riyadh, Saudi Arabia; e MYO Osteopathy, Riyadh, Saudi Arabia; f University College of Osteopathy, London, UK; g Instituto Piaget, Lisbon, Portugal; h Department of Psychology and Milan Center for Neuroscience (NeuroMI), University of Milano-Bicocca, Milan, Italy; i Research Centre for Brain & Behaviour, School of Natural Sciences & Psychology, Liverpool John Moores University, UK; j Institute of Psychology, Health & Society, University of Liverpool, UK

**Keywords:** Preterm, Infant, Affective, Touch, C-tactile, Heart-rate

## Abstract

Preterm birth is a significant risk factor for a range of long-term health problems and developmental disabilities. Though touch plays a central role in many perinatal care strategies, the neurobiological basis of these approaches is seldom considered. C-Tactile afferents (CTs) are a class of unmyelinated nerve fibre activated by low force, dynamic touch. Consistent with an interoceptive function, touch specifically targeted to activate CTs activates posterior insular cortex and has been reported to reduce autonomic arousal. The present study compared the effect of 5 min of CT optimal velocity stroking touch to 5 min of static touch on the heart-rate and oxygen saturation levels of preterm infants between 28- & 37-weeks gestational age. CT touch produced a significant decrease in infants’ heart-rates and increase in their blood oxygenation levels, which sustained throughout a 5-min post-touch period. In contrast, there was no significant change in heart-rate or blood oxygenation levels of infants receiving static touch. These findings provide support for the hypothesis that CTs signal the affective quality of nurturing touch, providing a neurobiological substrate for the apparent beneficial effects of neonatal tactile interventions and offering insight for their optimisation.

## Highlights

• Gentle, dynamic touch plays a central role in many perinatal care strategies.
• C-tactile afferents, unmyelinated mechanoreceptors, respond optimally to low force and velocity touch.
• CT targeted produced a significant decrease in infants’ heart-rates and increase in their blood oxygenation levels.
• Static touch did not generate significant change in heart-rate or blood oxygenation levels.
• Findings from this study provide support for the hypothesis that CTs signal the affective quality of nurturing touch.

## 1 Introduction

The World Health Organisation estimates that globally 15 million infants are born premature each year. While in high-income countries, as a result of advances in neonatal care, survival rates are increasing, long term physical and mental health problems as well as developmental disabilities are common (Wong et al., 2016; World Health Organization, 2016). The mechanisms underlying neurodevelopmental delay, reduced neural growth and abnormal neurofunctional organisation associated with preterm birth are currently poorly understood (e.g. Maitre et al., 2017; Malik et al., 2013; Nagy et al., 2003). Thus, a greater understanding of the effects of being in the Neonatal Intensive Care Unit (NICU) during a critical period of rapid neurodevelopment is required to optimise long-term outcomes (Als and McAnulty, 2011; McGlone et al., 2017; Pineda et al., 2014; Silbereis et al., 2016).

In utero, an infant receives continual multisensory input (Montagu, 1978). Indeed, stimulation of the maturing sensory systems is thought to contribute to the structural and functional development of the nervous system (Lecanuet and Schaal, 1996). The somatosensory system is the first to develop, from 8 weeks gestational age (GA) (Humphrey, 1964), with fetuses responding strongly to touch to the mother’s abdomen in the third trimester (Marx and Nagy, 2015). It has been hypothesised that activation of sensory nerves in the skin of the foetus by the amniotic fluid during movements is a mechanism underlying growth regulation (Bystrova, 2009). While in the incubator this cutaneous stimulation is absent, touch plays a central role in a range of perinatal care strategies including massage therapy, kangaroo care and osteopathic manipulative treatment (Als and McAnulty, 2011; Boundy et al., 2016; Cerritelli et al., 2013; Field, 2016; Gallace and Spence, 2010; Lanaro et al., 2017). Although the neurobiological basis of these interventions is not widely considered, and concerns around the methodological quality of studies used to assess them have been raised (e.g. Vickers et al., 2004), they do appear to have some positive effects on survival and growth (Boundy et al., 2016; Field, 2016). For example, kangaroo care, where the infant is held in skin-to-skin contact with the parent, consistently reduces neonatal morbidity and mortality (Boundy et al., 2016; Conde-Agudelo et al., 2014; Conde-Aguedelo and Díaz-rossello, 2016). Massage therapy too has reliably been reported to increase weight gain and reduce length of hospital stay in pre-term infants (Field, 2016). Furthermore, though follow up studies are rare, several have reported beneficial effects of massage on preterm infants’ cognitive development at both 12 and 24 months corrected age (Abdallah et al., 2013; Procianoy et al., 2010). Considering the specific type of touch that is beneficial, it is noteworthy that a review of the efficacy of massage interventions in preterm infants concluded babies who received dynamic but not static touch showed improvements in weight gain and reductions in length of hospital stay compared to those who were not touched (Vickers et al., 2004).

Whereas the discriminative properties of touch are signaled by thickly myelinated fast conducting Aβ afferents which project to primary somatosensory cortex, the affective components of touch are conveyed by a subclass of unmyelinated, slowly conducting C-type fibres named C-Tactile afferents (CTs) which project to limbic structures such as the insular cortex (McGlone et al., 2014; Vallbo et al., 1999). CT afferents are force, velocity & temperature tuned, responding preferentially to a gentle, stroking, skin temperature touch of between 1–10 cm/sec (Ackerley et al., 2014; Löken et al., 2009). This type of touch resembles a typical human caress (Morrison et al., 2010). Evidence for the biological relevance of this mechanosensory input comes from an observational study where, when asked to caress their infant, parents spontaneously delivered touch within the CT optimal range (Croy et al., 2016). Furthermore, a recent study reported that in 2-month-old infants, as in adults, greater activation was elicited in the insular cortex in response to CT optimal than to faster non-CT optimal touch, (Jönsson et al., 2018). Indeed, in term-infants just a few days old, gentle stroking has been found to lead to activation in the posterior insular cortex, consistent with the CT system being active in early infancy and thus able to influence early brain development (Tuulari et al., 2017).

Low intensity stimulation of cutaneous somatosensory nerves, through stroking touch, warmth and light pressure, has been reported to induce the release of endogenous peptides, such as oxytocin and opioids, and reduce arousal (Nummenmaa et al., 2016; Uvnäs-Moberg et al., 2014, 1996). Given their response characteristics, it seems likely activation of CTs plays a direct and significant role in these physiological effects (McGlone et al., 2017; Walker et al., 2017; Walker and McGlone, 2013). In support for this assertion, a number of recent behavioural studies reported that a brief touch, delivered at CT optimal velocity, to both adults and 9-month old infants, produced a significant reduction in heart-rate in comparison to slower and faster, non-CT optimal touch (Fairhurst et al., 2014; Pawling et al., 2017b, 2017a). However, these studies all used event-based designs where changes in heart-rate were measured over seconds, the velocity dependent effects of more sustained periods of touch are yet to be examined. While in a clinical setting brief tactile interventions in premature infants have been reported to produce immediate positive physiological effects, overall study methods, including timing and nature of touch, are inconsistent and findings mixed (Harrison, 2001; Vickers et al., 2004).

Thus, the aim of the present study was to test the hypothesis that preterm infants receiving 5 min of CT targeted stroking touch would show a greater reduction in physiological arousal than preterm-infants receiving static, non-CT optimal touch. To do this we used pulse oximetry to measure infants’ heart-rate and blood oxygen saturation levels, two frequently used physiological indicators of clinical outcome in newborn infants. Stressors, such as pain, cause an increase in heart-rate and blood pressure, a decrease in oxygen saturation, and more rapid, shallow, or irregular breathing (Sweet and McGrath, 1998). In contrast, a persistent or deep reduction in heart rate which positively influences cardiac output will have a beneficial effect on tissue oxygenation, as measured by peripheral capillary oxygen saturation (SpO2). Indeed, the two measures have previously been reported to show cross-correlation in premature infants with diverse clinical conditions (Fairchild and Lake, 2018; Fairchild et al., 2017).

## 2 Materials & methods

### 2.1 Participants

Ninety-two preterm infants (44 male), mean (±S.D.) gestational age 33.4 weeks (± 4.0) and mean weight at birth 2100 g (± 874) were recruited from the Neonatal Intensive Care Unit (NICU) of the Buzzi Hospital in Milan, Italy, from 6th January 2018 to 15th June 2018.

To be included in the study infants must be born in the Buzzi Hospital, with a gestational age (GA) between 28.0 and 36.6 weeks and without clinical (i.e. respiratory and cardiac distress) and/or congenital complications or suspected infections. All infants were enrolled within 1 week of birth.

Written informed consent was obtained from parents or guardians prior to an infant’s enrolment in the study. The study was approved by the Research Ethics Committee of Fatebenefratelli Hospital Group in Milan, Italy. All infants continued receiving routine neonatal clinical care for the duration of their enrolment in the study.

### 2.2 Design

Enrolled preterm infants were randomly allocated to two groups: (1) Dynamic Touch (2) Static Touch. The randomization sequence was computer-generated in blocks of ten, without stratification.

### 2.3 Physiological monitoring

Oxygenation and heart-rate were monitored with a pulse oximeter (Masimo corporation, Irvine, CA, USA). The paediatric pulse oximetry probe was attached around the dorsal aspect of the infant’s right foot. The physiological signals were digitized and recorded at 200 Hz using the New Life Box physiological recording system (Advanced Life Diagnostics, Weener, Germany) with Polybench software (Advanced Life Diagnostics, Weener, Germany).

### 2.4 Tactile stimulation procedure

All preterm infants underwent a single 15-min protocol, including delivery of either Dynamic or Static Touch. The interventions were delivered by researchers with experience in the field of neonatology.

Prior to data collection, the researchers underwent purposely designed training which focused on practicing the delivery of the dynamic stroking touch with consistent force and velocity (Pawling et al., 2017a). During training the researchers were guided in the delivering a consistent stroking velocity of 3 cm/sec by a visual metronome that moves across the same length of a computer screen as the area of skin to be stroked (10 cm) at the correct speed (3 cm/sec) (Etzi et al., 2018; Etzi and Gallace, 2016; Pawling et al., 2017a). The metronome also guided the researcher in the appropriate duration of the tactile stimulation. Open source software Psychopy (Pierce, 2009) was used to programme the visual metronome.

**Fig. 1:**
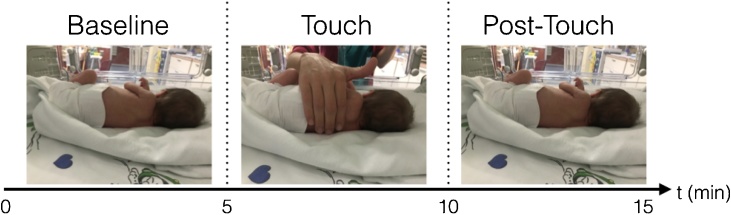
Timeline of the experiment, showing the position of all infants throughout the intervention and representative positioning of the experimenter’s hand during the static touch condition.

Each test session was composed of: a) 5-min Pre-Touch Baseline recording, b) 5-min Touch Procedure, c) 5-min Post-Touch recording (see Fig. 1). Touch was delivered using the researcher’s dominant hand. In order to match the skin temperature of the infant, the researcher placed their hand inside the incubator for the duration of the baseline period.

During the intervention, the area of stimulation was the dorsum of the infant, from the first thoracic to the last lumbar vertebra, covering approximately 10 cm. The contact was always bare hand to bare skin. During the full duration of the intervention, all infants were laid on their right side in the incubator. This position was chosen for clinical reasons as it was considered the optimal position to avoid possible obstruction from attached tubes and probes. Infants were placed in this position approximately 2 min before the recording started, with the pulse oximetry probe already attached to the right foot.

For infants in the Dynamic Touch Group, light stroking touch was applied at a velocity of approximately 3 cm/sec and an approximate force of 0.3 N. Dynamic stroking was delivered from the first thoracic to the last lumbar vertebra and in a reverse direction continuously, for 5 min.

For infants in the Static Touch Group, the researcher lightly placed their hand on the dorsum of the infant covering the area from the first thoracic to the last lumbar vertebra. The hand was kept in that position for the duration of the 5-min block, maintaining the same approximate force as in the Dynamic Touch condition (approx. 0.3 N).

All maneuvers were performed with constant fraction of inspired oxygen (FiO2) during the whole session. If any pharmacological administration was needed, i.e. caffeine, as per routine clinical care, it was performed at least 3 h before the session.

### 2.5 Data analysis

Physiology data was output as a CSV file with a data point for every 5 s of recording (200 Hz sampling frequency). On an individual participant basis, oxygenation and heart-rate data were averaged into 30-s-long time bins and divided into Baseline, Touch and Post-Touch Periods i.e. 10 data points per time block. Pulse oximeters can be sensitive to movement artifacts therefore, to account for non-experimental movements that might produce extreme values, individual participant’s Baselines containing datapoints that were more than three S.D.s above or below the whole sample mean were identified. Such datapoints were determined to be artefacts and replaced by the mean of that participant’s non-artifactual epochs from the Baseline period.

Considering the peripheral capillary oxygen saturation (SpO2), 5 participants were identified as having artifactual epochs in the Baseline period, across participants this represented 2.39% of Baseline period datapoints. For HR, 3 participants had artifactual epochs, across participants this represented 0.87% of datapoints from the Baseline period.

Subsequently, for both oxygenation and heart-rate data, each data point from the Touch and Post-touch periods were converted to a change from Baseline, by subtracting that participant’s mean Baseline from each of the 20 epochs in the Touch and Post-Touch periods. Again, on a participant by participant basis, data points were identified which lay more than 3 SDs outside the grand mean for the sample on each of the 2 measures and artifactual epochs were replaced with the mean of that participant’s non-artifactual epochs within a given time period.

Considering SpO2, 5 participants in the Touch period and 5 in the Post-Touch period were identified as having artifactual epochs, across participants this reflected 1.85% of datapoints in each of the Touch and Post-Touch periods. For the HR, 4 subjects in the Touch period and 3 in the Post-Touch period were identified as having artifactual epochs, reflecting 0.76% and 0.54% of datapoints in the Touch and Post-Touch periods respectively.

There were no significant differences in the percentage of artifactual epochs identified in either the HR or SpO2 data between the two groups (Dynamic Touch, Static Touch) or across the three time periods (Baseline, Touch, Post-Touch), all F’s <1.

Two participants, one from each group, were excluded from the SpO2 analysis as they had no non-artifactual data points in one of the 3 time periods. However, they were both included in the heart-rate analysis.

Data were analysed in SPSS (Version 23, IBM Corp, USA, NY). To explore differences between groups over time, change scores for heart-rate and oxygenation were entered into separate Repeated Measures ANOVAs with the following factors: Group (Dynamic Touch vs Static Touch) and Epoch (20 * 30 s time bins).

Subsequently data were collapsed into two datapoints, by calculating the mean heart-rate and oxygenation for the Touch and Post-Touch periods. Then the data from the HR and SpO2 were analysed using separate repeated measures ANOVAs with the factors of Group (Dynamic Touch vs Static Touch) and Time (Touch and Post-Touch). Single sample T-tests were used to determine whether changes were significantly different from Baseline.

General characteristics of the Dynamic and Static Touch groups, GA, Weight at Birth and Baseline heart-rate and oxygenation were compared with independent samples t-tests. Gender Distribution between groups was compared using a Chi Square test.

## 3 Results

### 3.1 Sample characteristics & baseline measures

As shown in Table 1, the two groups were homogenous at Baseline in terms of GA, weight at birth, gender distribution, heart-rate and oxygenation levels.

**Table 1:** General characteristics of the study population at baseline. Values shown are mean ± S.D. All P values are from t tests except Gender* N(%), p values from X^2^.

	Dynamic Touch (n = 46)	Static Touch (n = 46)	p>|t|
Gestational age (wk)	32.8 ± 4.0	33.9 ± 4.1	0.18
Weight at birth (g)	2027 ± 799	2173 ± 948	0.43
Gender*	23 (50)	21 (46)	0.8
Heart-rate	145.9 ± 17.5	145.6 ± 11.3	0.84
SpO2	96.1 ± 3.6	96.1 ± 4.4	0.99

### 3.2 Heart-rate

**Fig. 2:**
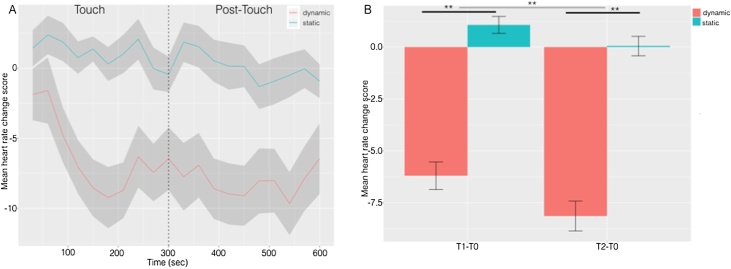
**A)** The time course in seconds of heart-rate in response to Dynamic and Static touch during the 5-min-long Touch & Post-Touch periods. Data are presented as change in beats-per-minute from Baseline for 10*30-s-long epochs in each period. The shaded area represents +/− 1 S.E. **B)** Bar-chart displaying the mean heart-rate recorded in the Dynamic and Static touch conditions during the Touch and Post-Touch periods. Again, data are presented as change in beats per-minute from Baseline. Error bars show +/− 1 S.E. Black lines indicate the significant effect of Group. The grey line indicates the significant effect of Time. **p < 0.01.

There was a significant effect of Epoch *F*(19,1710) = 3.831, *p* < 0.001 partial η^2^ = 0.041, as well as of Group *F*(1,90) = 12.86, *p* = 0.001 η^2^ = 0.125 and a Group x Epoch interaction *F*(19,1710) = 1.612, *p* = 0.046, partial η^2^ = 0.18. As can be seen in Fig. 2A, the Dynamic Group showed a reduction in heart-rate early in the Touch Period, which was maintained throughout the Post-Touch period. However, the Static Touch group did not show this effect.

To explore the observed group difference further, data were collapsed into two data points, by calculating means for the Touch and Post-Touch period. As illustrated in Fig. 2B, there was a significant effect of Time *F*(1,90) = 7.73, *p* = 0.007, partial η^2^ = 0.79, reflecting a lower heart-rate, on average in the Post-Touch than the Touch period. In addition, there was significant effect of Group *F*(1,90) = 12.86, *p* = 0.001, partial η^2^ = 0.125 but no Group x Time interaction (F < 1).

Post-hoc single sample T-tests revealed that the group receiving Dynamic Touch showed a significant reduction in heart-rate from Baseline during both the Touch and Post-Touch periods, *t*(45) = 3.531, *p* = 0.001; *t*(45) = 4.03, *p* = 0.0001. Whereas, the group receiving Static Touch showed no significant change in heart-rate in comparison to Baseline in either period, *t*(45) = 1.066, *p* = 0.29; *t*(45) = 0.02, *p* = 0.98

### 3.3 Oxygen saturation

**Fig. 3:**
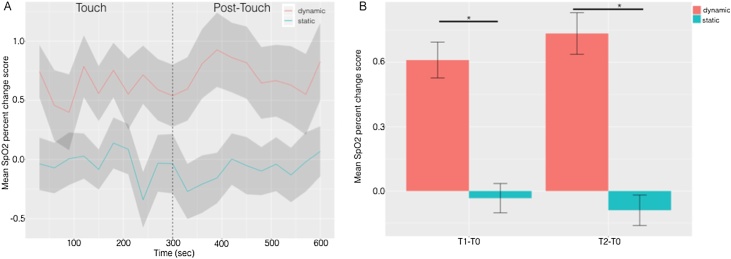
**A)** The time course in seconds of blood oxygen-saturation levels (%) in response to Dynamic and Static touch during the 5-min-long Touch & Post-Touch periods. Data are presented as change in percentage saturation from Baseline for 10*30-s-long epochs in each period. The shaded area represents +/− 1 S.E. **B)** Bar-chart displaying the mean blood oxygen-saturation level recorded in the Dynamic and Static touch conditions during the Touch and Post-Touch periods. Again, data are presented as change in percentage saturation from Baseline. Error bars show +/− 1 S.E. Black lines indicate the significant effect of Group. *p < 0.05.

For SpO2, there was a significant effect of Group *F*(1,88) = 6.88, p = 0.01 η^2^ = 0.073 but not of Epoch, nor was there a significant Group x Epoch interaction (Fs < 1). See Fig. 3A.

The second analysis, using the mean change in oxygen saturation from Baseline in both the Touch and Post-Touch periods, revealed a significant main effect of Group *F*(1,88) = 6.89, *p* = 0.01, patial η^2^ = 0.73 but again no significant effect of Time nor a Group x Time interaction (Fs < 1), see Fig. 3B. Post hoc single sample T-tests demonstrated that the Dynamic Group showed a significant increase in oxygenation compared to Baseline in both the Touch and Post-Touch periods: *t*(44) = 3.06, *p* = 0.004; *t*(44) = 2.88, *p* = 0.006, whereas the Static Group showed no significant change from baseline oxygenation levels at either time point, *t*(44) = 0.2, *p* = 0.85; *t*(44) = 0.46, *p* = 0.65.

## 4 Discussion

The results of the present study show that a short period of dynamic stroking touch, delivered at a force and velocity to optimally activate CTs, produces a reduction in the heart-rate of preterm infants that was sustained into a 5-min post-touch period. In contrast, static touch, which would not activate CTs as strongly, produced no significant change in the heart-rate of preterm infants matched for weight and gestational age. Dynamic touch was also associated with an increase in levels of oxygen saturation, which was not seen in those infants receiving static touch. These findings are consistent with our previous studies in adults, and another in 9-month-old infants, which report that brief periods of CT optimal velocity stroking touch reduce heart rate to a significantly greater extent than faster or slower velocity touch (Fairhurst et al., 2014; Pawling et al., 2017a, 2017b). The present study extends this previous work by examining effects over an extended time period. That is, while previous studies have used event-based designs with changes in heart-rate being observed over seconds, here we report that not only are heart-rate changes observed in response to a more sustained period of dynamic touch (5 min) but that the effects are maintained beyond the period of touch stimulation. We interpret the accompanying increase in blood oxygen saturation as reflecting the positive influence of this heart rate change on infants’ physiological state (Sweet and McGrath, 1998). Our findings are also consistent with several recent studies that indicate the CT system is functionally mature in early infancy (Jönsson et al., 2018; Tuulari et al., 2017). Here we show these differential responses to CT optimal and non-optimal touch are also present in preterm infants younger than 40 weeks GA.

These findings are consistent with previous reports that cutaneous stimulation, through dynamic stroking or massage, results in changes in autonomic and endocrine function. For example, reducing heart-rate and blood pressure, and increasing high frequency heart rate variability (Diego et al., 2014, 2004, 2005; Field et al., 2006; Lund et al., 1999). Indeed, increases in vagal activity leading to greater gastric motility are hypothesized to underpin the increased weight gain observed in pre-term infants receiving repeated massage interventions (Diego et al., 2014, 2007, 2005).

Further support for our hypothesis that CTs are the cutaneous nerves underpinning these effects comes from comparison of the physiological and behavioral effects of CT targeted skin stimulation with those of oxytocin, release of which can be induced by low intensity cutaneous stimulation (Uvnäs-Moberg et al., 2014; Walker et al., 2017). For example, oxytocin reduces hypothalamic–pituitary–adrenal (HPA) axis-activity and increases pain thresholds (Paloyelis et al., 2016; Rash et al., 2014; Uvnäs-Moberg et al., 2015; Walker et al., 2017). Similarly, a sustained period of CT targeted stroking touch has been reported to increase heart rate variability (Triscoli et al., 2017) and decrease neural and behavioural responses to noxious painful stimuli in both adults and infants (Gursul et al., 2018; Habig et al., 2017; Krahé et al., 2016; Liljencrantz et al., 2017). While we did not include a measure of endocrine function in the present study, it has previously been reported that 5 min of CT optimal stroking touch administered to the dorsum of conscious rats resulted in an increased selective activation of hypothalamic oxytocin neurons compared to the same period of static touch (Okabe et al., 2015).

The findings of the present study are also consistent with a previous review of infant massage interventions which concluded that dynamic, rather than static, touch was needed to deliver improvements in weight gain and reductions in length of hospital stay (Vickers et al., 2004). However, the fact we observed autonomic consequences with a relatively low force touch is inconsistent with several previous studies reporting that medium pressure touch, “sufficient to produce a slight indentation in the skin”, is required to induce short-term reductions in heart rate and longer-term benefits, such as weight gain in pre-terms (Diego et al., 2004; Field et al., 2006). However, closer inspection of the data reported in Diego et al (2004), shows that mean heart rate during touch did not differ between light and moderate pressure conditions. Rather, higher levels of variance and lower mean baseline heart rate in the light touch condition explain the group differences reported (Diego et al., 2004 Fig. 2). Thus, further research is required to determine the acute effects of different forces of cutaneous stimulation on autonomic function.

The force used in the present study was identified in previous microneurography studies to be sufficient to activate CTs (Löken et al., 2009). It was also consistent with previous studies from our group where stroking touch was delivered manually (Etzi et al., 2018; Pawling et al., 2017a). While CTs are low threshold mechanoreceptors, responding to much lighter forces of stimulation than high threshold mechanoreceptors coding nociceptive inputs, they do not respond exclusively to low forces, and will maintain their firing frequency as mechanical force increases (Vallbo et al., 1999). In contrast, high threshold mechanoreceptors increase their firing frequency with increasing force. Thus, CTs will respond to the same extent to both light and moderate force touch, so any difference in the long and short term physiological effects of light and moderate massage are unlikely to be due to the differential responding of CTs. It is more likely that other classes of mechanosensory afferent and or baroreceptors underpin the reported differential effects.

The lack of effect of static touch in the present study needs to be reconciled with the previously reported benefits of skin-to-skin contact, such as kangaroo care, on preterm infants’ growth and development (Bergman et al., 2004; Boju et al., 2011; Feldman and Eidelman, 2003). Infants’ Respiratory Sinus Arrhythmia (an index of parasympathetic arousal) levels have been found to adjust to those of their mother’s when lying on her chest (Bloch-Salisbury et al., 2014; Van Puyvelde et al., 2015). The effect is thought to be mediated by the mother’s cardiac rhythm and is an extension of the transfer effects that occur in utero (Van Leeuwen et al., 2009). However, the static touch of a hand provided in the present study does not allow for these cardio-respiratory mediated effects to be mirrored. Rather, the beneficial effects of dynamic touch reported here provide additional support for the hypothesis that activation of CTs, as occurs with dynamic touch and which mothers have been shown to deliver intuitively, provides another mechanism for supporting infants’ physiological regulation (Van Puyvelde et al., 2019).

The present study examined only the acute effects of a brief touch intervention on short-term measures of heart rate and blood oxygenation. Future work is needed to determine whether there are longer-term, clinically significant, benefits to this type of touch as an intervention and if so what dose, in terms of frequency and duration, is required (McGlone et al., 2017). Systematic assessment of the effects of infant massage interventions by Field and colleagues have determined that 15 min of massage 3 times per day for 5 days is sufficient to increase weight gain (Diego et al., 2014; Field et al., 2006). In their massage protocol, the baby is massaged in different body areas, moving from head to limbs, over the 15-min intervention. In our present study, we concentrated on the back as previous genetic visualization studies in rodents (Liu et al., 2007), and our own psychophysical data from adult humans (Walker et al., 2016), indicate CTs may innervate this area most densely. However, CTs do show fatigue, represented by a reduced firing rate to repeated tactile stimuli such as delivered in the present study (Vallbo et al., 1999). Therefore, comparison of the physiological effects of the same period of CT targeted touch delivered to a single versus a range of body sites would be insightful.

It is noteworthy that Diego et al. (2004) used a hypoallergenic baby oil in their massage protocol to reduce friction. The effects of topical skin treatments on the responses of CTs are not known, however it is possible that with reduced friction, applied forces are dissipated, thereby recruiting more CT terminals. Further microneurography studies, systematically comparing the response properties of CT afferents when materials with different rheological properties are applied to the skin are needed to address this question.

In conclusion, tactile input is known to have a significant impact both pre and postnatally on the developing infant (McGlone et al., 2017; Sharp et al., 2012; Whitehead et al., 2018). While touch based interventions can have some clinical benefits for pre-term infants, greater understanding of the neurobiological mechanisms underlying these effects is needed to optimize protocols and ameliorate the long-term negative consequences of pre-term birth on neural development and hence cognitive function. The present study supports the hypothesis that a specific class of cutaneous sensory afferents, which respond preferentially to low force, dynamic touch, delivered at skin temperature, are mediators of the beneficial autonomic consequences of neonatal tactile interventions. Offering insight for the development and optimisation of novel perinatal care strategies.

## Funding

This research did not receive any specific grant from funding agencies in the public, commercial, or not-for-profit sectors.
